# Muscle-related parameters-based machine learning model for predicting postinduction hypotension in patients undergoing colorectal tumor resection surgery

**DOI:** 10.3389/fmed.2023.1283503

**Published:** 2023-12-27

**Authors:** Weixuan Sheng, Danyang Gao, Pengfei Liu, Mingxue Song, Lei Liu, Huihui Miao, Tianzuo Li

**Affiliations:** ^1^Department of Anesthesiology, Beijing Shijitan Hospital, Capital Medical University, Beijing, China; ^2^Department of Science and Technology, Beijing Shijitan Hospital, Capital Medical University, Beijing, China

**Keywords:** postinduction hypotension, machine learning, Boruta, regression fitting curve, partial dependence profile

## Abstract

**Objectives:**

This study used machine learning algorithms to identify important variables and predict postinduction hypotension (PIH) in patients undergoing colorectal tumor resection surgery.

**Methods:**

Data from 318 patients who underwent colorectal tumor resection under general anesthesia were analyzed. The training and test sets are divided based on the timeline. The Boruta algorithm was used to screen relevant basic characteristic variables and establish a model for the training set. Four models, regression tree, K-nearest neighbor, neural network, and random forest (RF), were built using repeated cross-validation and hyperparameter optimization. The best model was selected, and a sorting chart of the feature variables, a univariate partial dependency profile, and a breakdown profile were drawn. R^2^, mean absolute error (MAE), mean squared error (MSE), and root MSE (RMSE) were used to plot regression fitting curves for the training and test sets.

**Results:**

The basic feature variables associated with the Boruta screening were age, sex, body mass index, L3 skeletal muscle index, and HUAC. In the optimal RF model, R^2^ was 0.7708 and 0.7591, MAE was 0.0483 and 0.0408, MSE was 0.0038 and 0.0028, and RMSE was 0.0623 and 0.0534 for the training and test sets, respectively.

**Conclusion:**

A high-performance algorithm was established and validated to demonstrate the degree of change in blood pressure after induction to control important characteristic variables and reduce PIH occurrence.

## 1 Background

Postinduction hypotension (PIH) is a common complication that occurs during the induction of general anesthesia in clinical practice. Because the arterial blood pressure is lower than the lower limit of the vascular autoregulation curve, it may lead to ischemia in important organs (such as the heart, brain, and kidneys) (1). Many clinical studies have shown an association between PIH and organ dysfunction, including postoperative acute kidney injury, myocardial injury, and postoperative 30-day mortality (2, 3). Previous studies have shown that PIH is caused by multiple factors; however, the exact pathophysiological mechanisms are not fully understood. PIH is induced by multiple factors, such as the patient’s physical condition and anesthesia management (4).

Malnutrition is the main risk factor for adverse perioperative outcomes. Patients undergoing gastrointestinal tumor surgery may present with varying degrees of malnutrition, sarcopenia, and myosteatosis (5). Sarcopenia refers to the gradual loss of skeletal muscle mass and strength and is a cause for concern because it may result in many adverse outcomes in older adults, including physical disability, poor quality of life, and increased mortality. Myosteatosis is an ectopic fat depot that increases and is negatively correlated with muscle mass, strength, and mobility, disrupting metabolism (insulin resistance and diabetes). Studies have shown that sarcopenia and myosteatosis are associated with an increased incidence rate, postoperative complications, and overall reduction in survival (6–8). Abdominal computed tomography (CT) can diagnose sarcopenia and myosteatosis, while muscle mass can reflect an individual’s tolerance to general anesthesia. We assumed that the parameters measured using CT might impact the incidence of PIH. Therefore, we collected preoperative clinical data and CT imaging parameters from patients undergoing colorectal cancer surgery to explore risk factors for PIH (9, 10).

In the past 5 years, only one systematic review has described the risk factors for PIH, and research has shown that aging, ASA, emergency surgery, low baseline blood volume, and long-term intake of ACEI/ARB, propofol, and fentanyl are risk factors for PIH, while weight gain is a protective factor (11). Therefore, accurately identifying high-risk factors for PIH and taking corresponding measures are effective methods to reduce its occurrence. In recent years, the use of artificial intelligence (AI) has increased rapidly in the medical field. Machine learning, as a major branch of AI, has the advantages of more stable model construction and more accurate prediction and is widely used in clinical prediction and other aspects. This study applied machine learning algorithms to determine risk factors of the degree of changes in blood pressure after induction and establish predictive models to assist clinicians in developing accurate, personalized management plans for patients in a timely manner.

## 2 Materials and methods

### 2.1 Research subjects and data collection

The Ethics Committee of Shijitan Hospital approved the study protocol and waived the requirement for informed consent. This single-center retrospective study obtained perioperative clinical data from the electronic medical records of 318 patients who underwent colorectal tumor resection surgery between September 1, 2018, and September 1, 2021.

The inclusion criteria were age > 18 years, abdominal surgery, and surgical duration of at least 1.5 h. The exclusion criteria were severe respiratory system diseases (pulmonary embolism, COPD or asthma), severe neurological diseases (cerebral infarction, cerebral hemorrhage or Parkinson’s disease), severe cardiovascular system diseases (NYHA III or IV, arrhythmia or hypertension grade III), severe liver and kidney dysfunction (Child-Pugh B, C level, CKD Phase II or above), and affected patients with abdominal CT findings.

### 2.2 Anesthesia management and evaluation of the outcome variable

The patient entered the room, and a peripheral vein was used to monitor heart rate, blood pressure, and blood oxygen saturation (SpO_2_). The bispectral index was measured with a bispectral EEG monitor. General anesthesia was induced after the infusion of 200–300 mL of the crystal liquid. General anesthesia was induced with remifentanil (TCI: Minto mode), with a target effect-site concentration (Cet) of 2 ng/mL, and the effect-site concentration was set to reach the same concentration as the TCI pump display. TCI induction was started with propofol, with Cet set to 2 μg/mL (TCI: improved Marsh mode). If induction was not achieved within 3 min, the Cet was gradually increased by 0.5 μg/mL every 30 s until the consciousness sedation score (OAA/S) was 0. Finally, rocuronium 0.6 mg/kg was administered for tracheal intubation. After induction intubation, target propofol (Cet 2–4 μg/mL) and remifentanil (Cet 3.0–6.0 ng/mL) were achieved, and BIS values were maintained at 40–60. Propofol and remifentanil pumping was stopped for all patients at the end of the surgery, and they were admitted to the PACU after surgery.

Postinduction blood pressure change rate is taken as the outcome variable, which is collected as a continuous variable and defined as the rate of decrease in average blood pressure compared to baseline. The time frame of PIH was between anesthesia induction and surgical incision or 20 min after induction, whichever occurred first (11). When the induced blood pressure was lower than the baseline, postinduction blood pressure change rate was defined as a positive value, whereas when the induced blood pressure was higher than the baseline, postinduction blood pressure change rate was defined as a negative value. When postinduction blood pressure change rate is greater than 20%, it is defined as PIH (11).

### 2.3 CT imaging measures

The patients’ cross-sectional CT scans were assessed at the level of the third lumbar vertebra (L3). The bilateral psoas major, paraspinal, and abdominal muscles were determined sequentially and summed to obtain the total SMA at the L3 level (total SMA, cm^2^). The skeletal muscle index (SMI, cm^2^/m^2^) was defined as the total SMA at the L3 level, standardized by patient height. Muscle quality was evaluated using the HU average calculation (HUAC) (12). The following equations were used: HUAC = [(RPHU*RPA) + (LPHU*LPA)] / (RPA + LPA) (RPHU: right psoas HU; RPA: right psoas area; LPHU: left psoas HU; LPA: left psoas area).

### 2.4 Characteristic variables

Characteristic variables included age, sex, body mass index (BMI), Hb, ASA grade, TNM grade, age-adjusted Charlson Comorbidity Index, prognostic nutritional index [= 10*serum albumin (g/dL) +0.005*total lymphocyte counts (/mm^3^)], L3 SMI, HUAC, and PIH.

### 2.5 Statistical analysis and sample size

R (version 4.2.2) and RStudio (version 2023.06.0 + 421) were used for statistical analysis. The normal distribution of numeric variables was tested using the Shapiro–Wilk test. Continuous variables with a normal distribution are presented as the mean ± standard deviation (SD). Continuous variables with a non-normal distribution are presented as the median (IQR). Categorical data are expressed as numbers (%). If the percentage of missing values was more than 20%, it was excluded from the final completed dataset. However, if it was less than 20%, the missForest package was used for interpolation (13).

Boruta was used to screen the relevant basic characteristic variables in the training set (14). After standardizing the data, four models, regression tree, K-nearest neighbor, neural network, and random forest (RF), were built using repeated cross-validation and hyperparameter optimization in the training set (15). Repetitive cross-validation divided the training set into 10 mutually exclusive subsets of the same size. One subset was used as a validation dataset for the model, whereas the other nine subsets were used to train the model. The appeal process was repeated 10 times. Thereafter, the best model was selected, and a sorting graph of the feature variables, a univariate partial dependency profile, and a breakdown profile were drawn. R^2^, mean absolute error (MAE), mean squared error (MSE), root mean squared error (RMSE), and regression fitting curves were obtained for the training and test sets (16–18). With the machine learning algorithms for regression models, six common supervised learning algorithms was chosen: regression tree (RT), K-nearest neighbor (KNN), support vector machine (SVM), neural network (NNET), extreme gradient enhancement (Xgboost), and random forest (RF). Among these, four of them was retained (RT, KNN, NNET, and RF) with relatively low MSE value.

The basic principle of the RF model is as follows:


$$Y=H\left(x\right)=\arg\max_{y}\overset{n}{\underset{k=1}{\sum }}I\left(h_{k}\left(x\right)=y\right)$$

H(x) is a combination classification model; Y is the final classification result; $h_{k}$ (x) is a single decision tree classifier; y is the classification result of a single decision tree classifier; and I (·) is an indicative function.

For the regression prediction model, the calculated final sample size obtained through the pmsampsize function of RStudio was 244, less than the sample size of 318 included in this study, with function parameters set as follows: adjusted maximum *R*^2^ = 0.7; number of independent variable parameters to be included = 10; average outcome of postinduction blood pressure change rate = 0.2; and SD of postinduction blood pressure change rate = 0.15 (19).

## 3 Results

### 3.1 Flowchart and baseline clinical data

**Figure 1:**
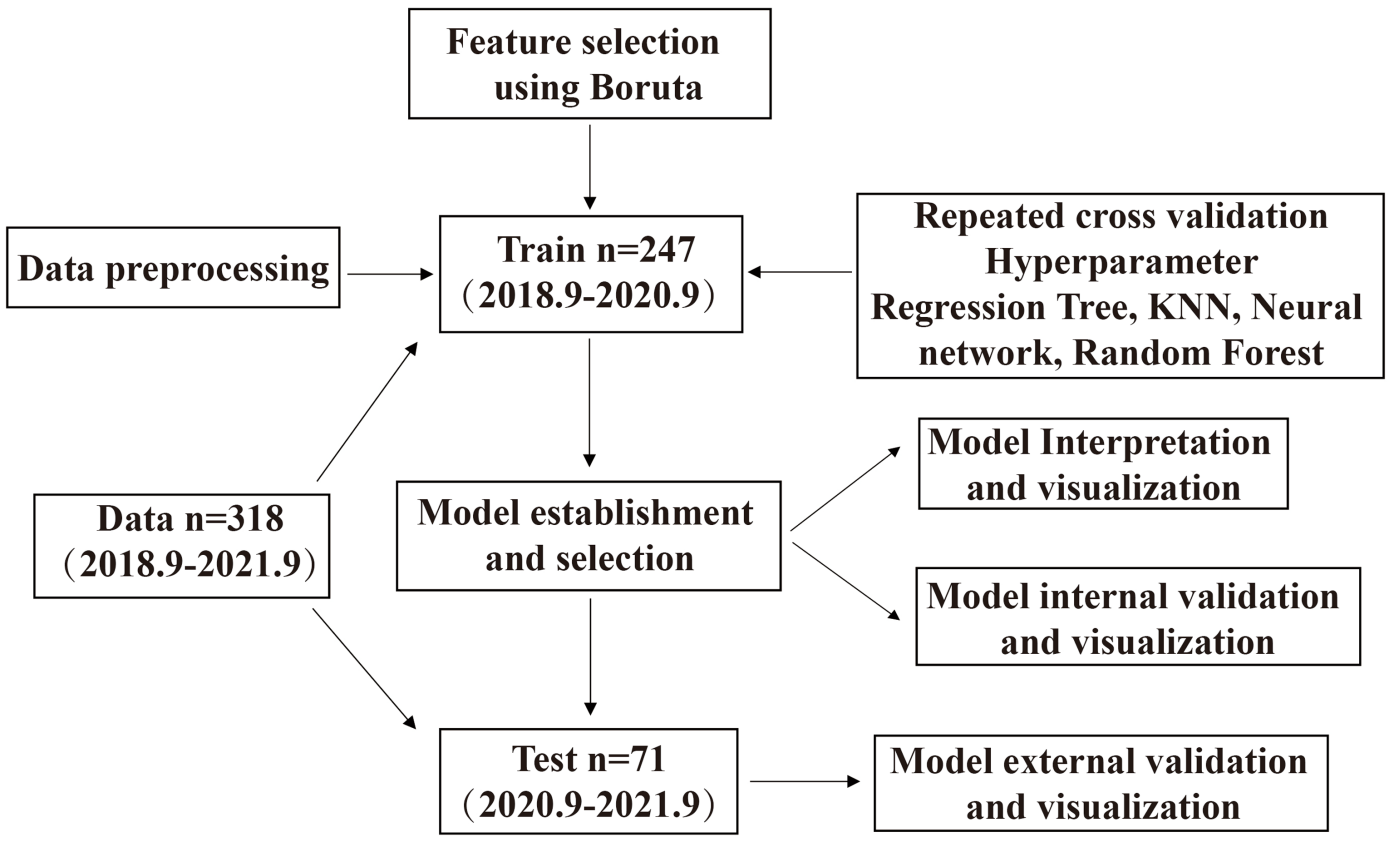
Flow chart of clinical data.

In total, 318 patients were included in the original study. The baseline parameters of the training and test sets are listed in Table 1. The incidence of PIH in our study was 59.92% (148/247) in the training set and 61.97% (44/71) in the testing set, as shown in Table 1. The processes of data inclusion, feature selection, model establishment, selection, visualization, and internal and external validation are shown in Figure 1. We collected clinical information from a total of 318 patients, including 10 independent variables and 1 dependent variable, totaling 11 variables. Therefore, we analyzed a total of 318 * 11 = 3,498 data.

**Table 1:** Baseline of clinical data.

Factor		Train (*n* = 247)	Diagram	Test (*n* = 71)	Diagram
Age (years)		66.00 (58.00, 72.00)	▁▃▆▇▂	66.00 (57.50, 71.00)	▁▅▇▆▂
BMI (kg/m^2^)		23.44 (21.26, 25.37)	▂▇▇▂▁	23.44 (21.14, 25.39)	▂▆▇▃▁
Hb (g/L)		124.0 (108.0, 137.0)	▂▃▇▇▂	122.0 (107.5, 139.5)	▃▃▇▆▅
aCCI		5.00 (4.00, 7.00)	▇▇▃▂▁	5.00 (4.00, 6.50)	▃▇▇▁▁
PNI		46.10 (42.25, 50.08)	▇▁▁▁▁	46.85 (42.50, 48.98)	▁▂▆▇▂
L3 SMI (cm^2^/m^2^)		41.30 (35.31, 47.72)	▃▇▆▂▁	43.46 (36.81, 49.87)	▅▇▇▆▂
HUAC		37.89 (33.72, 41.88)	▂▅▇▅▁	38.46 (34.15, 42.46)	▁▂▃▇▃
Postinduction blood pressure change rate		0.239 (0.156, 0.319)	▁▁▅▇▃	0.239 (0.166, 0.316)	▁▁▅▇▆
Gender	Male	159 (64.37)		47 (66.20)	
	Female	88 (35.63)		24 (33.80)	
ASA	ASA-I	2 (0.81)		0 (0)	
	ASA-II	173 (70.05)		49 (69.01)	
	ASA-III	71 (28.74)		22 (30.99)	
	ASA-IV	1 (0.40)		0 (0)	
TNM	I	36 (14.57)		10 (14.08)	
	II	90 (36.45)		19 (26.76)	
	III	79 (31.98)		29 (40.85)	
	IV	42 (17.00)		13 (18.31)	
PIH	YES	148 (59.92)		44 (61.97)	
	NO	99 (40.08)		27 (38.03)	

### 3.2 Feature variables selection using Boruta and model selection based on mse in the training set

**Figure 2:**
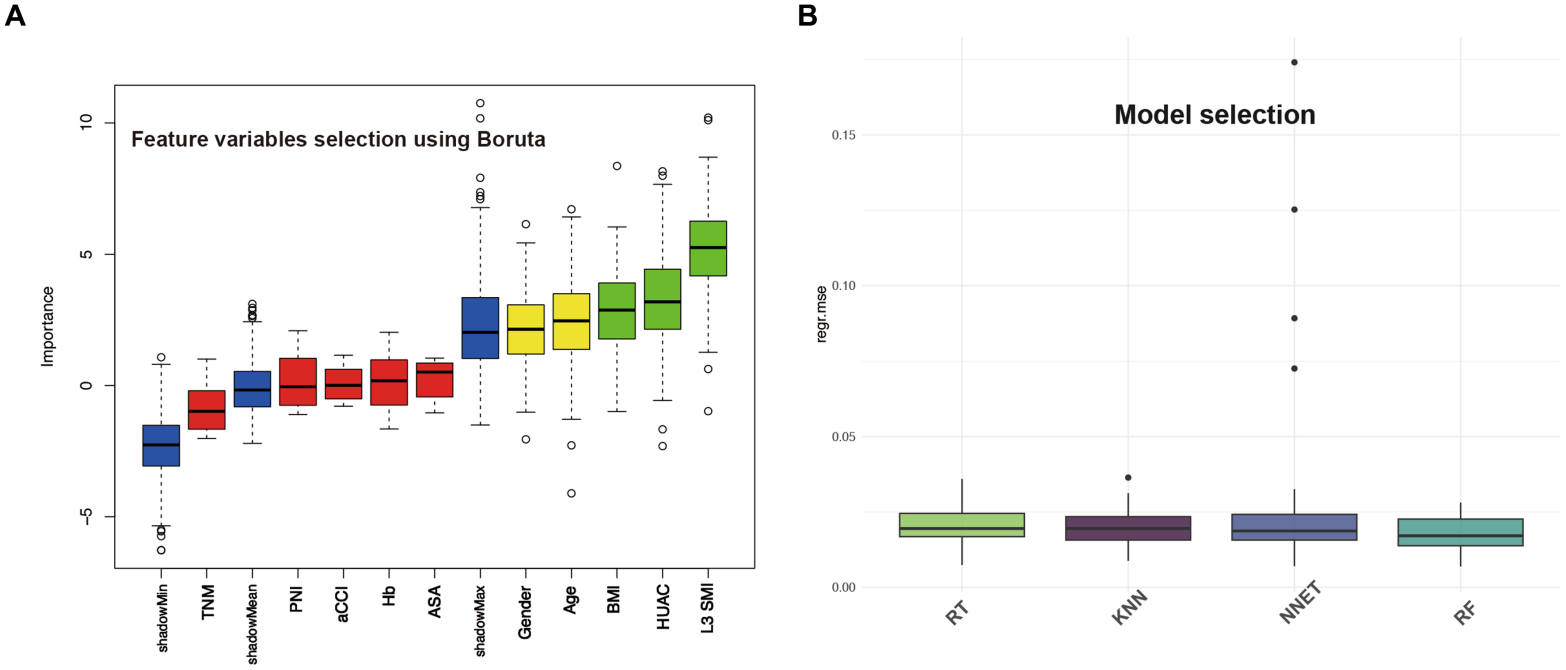
Feature variables selection using Boruta and model selection based on MSE.

Boruta analysis showed that age, sex, BMI, L3 SMI, and HUAC were the five feature variables included in the model (Figure 2A). Among the four models in this study, RF exhibited the lowest MSE, as shown in Figure 2B.

### 3.3 Regression fitting curves in the training and test set in RF

**Figure 3:**
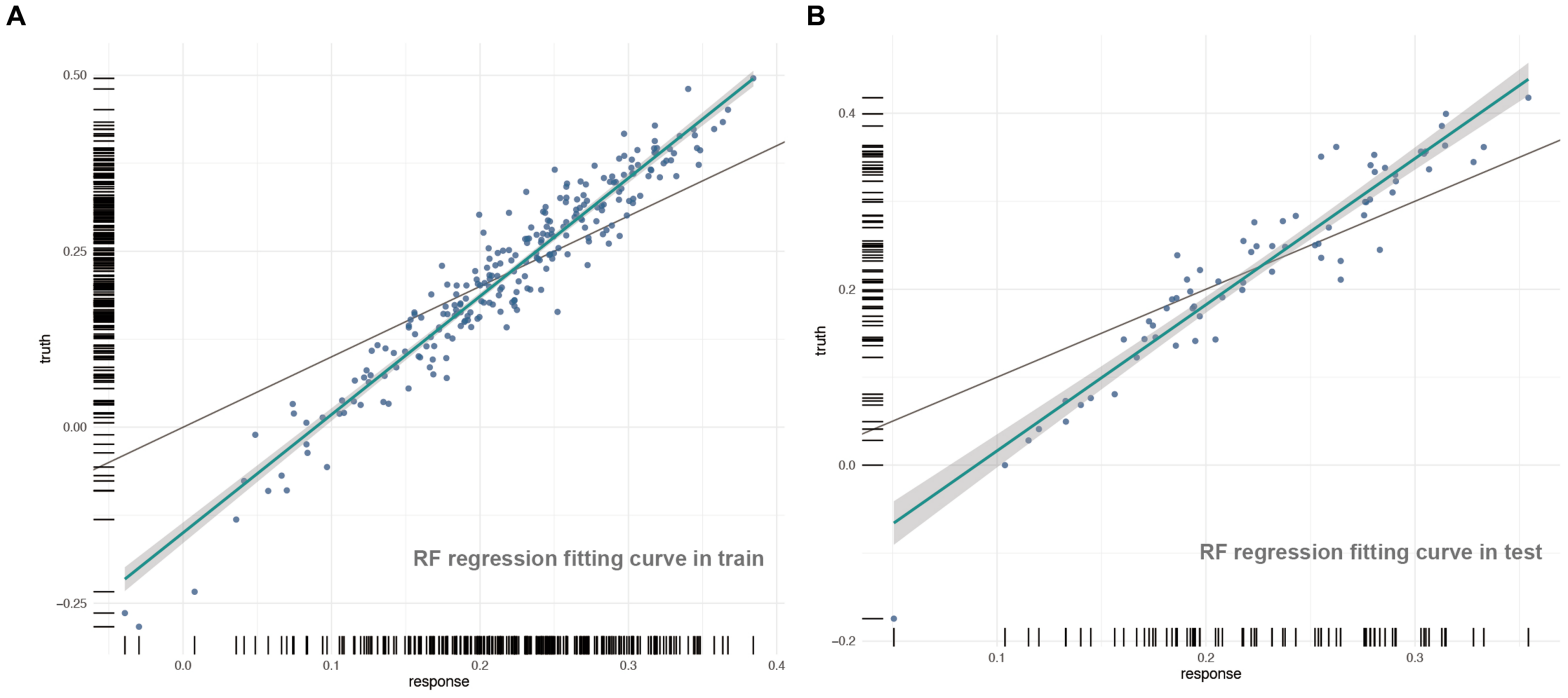
The regression fitting curves on train and test set in RF model.

In Figure 3, for the training (A) and test (B) sets, the green line represents the RF model fitting curve, and the gray line represents the benchmark curve. R^2^ was 0.7708 and 0.7591, MAE values were 0.0483 and 0.0408, MSE values were 0.0038 and 0.0028, and RMSE values were 0.0623 and 0.0534 for the training and test sets, respectively.

### 3.4 Sorting chart and univariate partial dependency profile of feature variables

**Figure 4:**
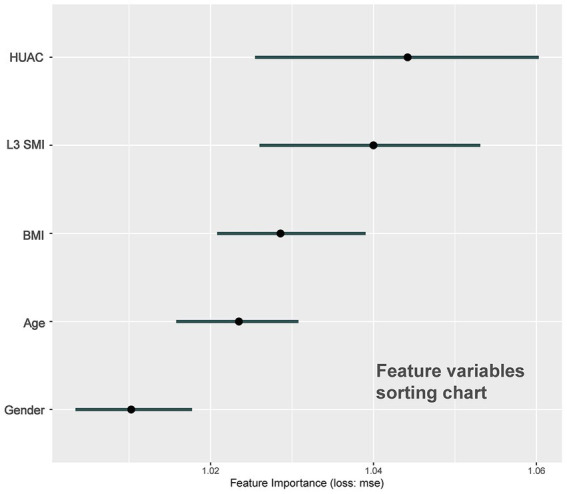
Sorting chart of feature variables.

**Figure 5:**
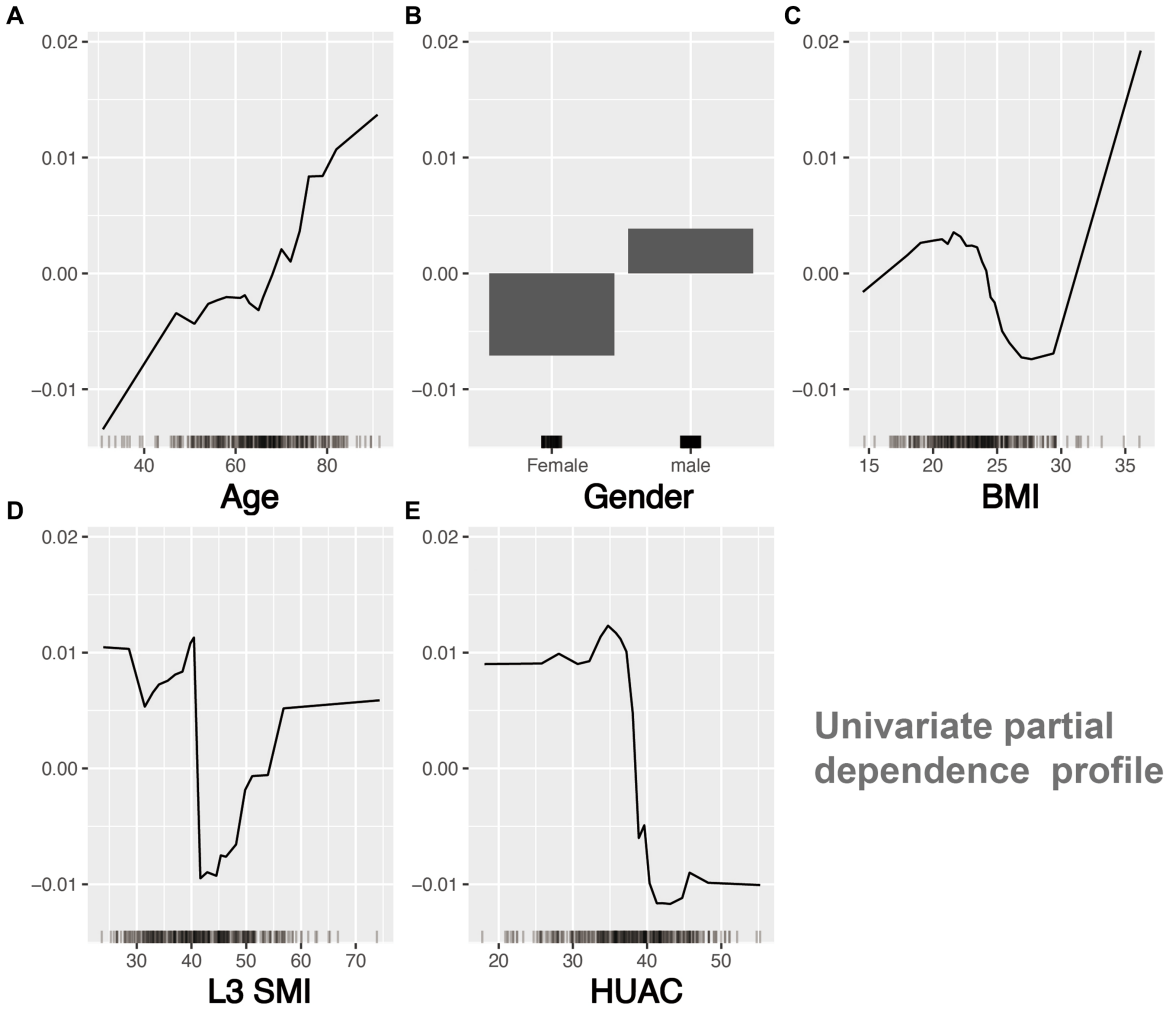
Univariate partial dependency profile of feature variables.

The sorting chart and partial dependency profiles of the five feature variables were established using the RF model (Figures 4, 5, respectively). In the importance ranking, we can intuitively determine how much each feature variable contributes to the predicted variable. The partial dependency profile was used to analyze the RF model, showing the impact of each feature in the sample and the changing trend of postinduction blood pressure with each feature variable.

### 3.5 Breakdown profile of RF

**Figure 6:**
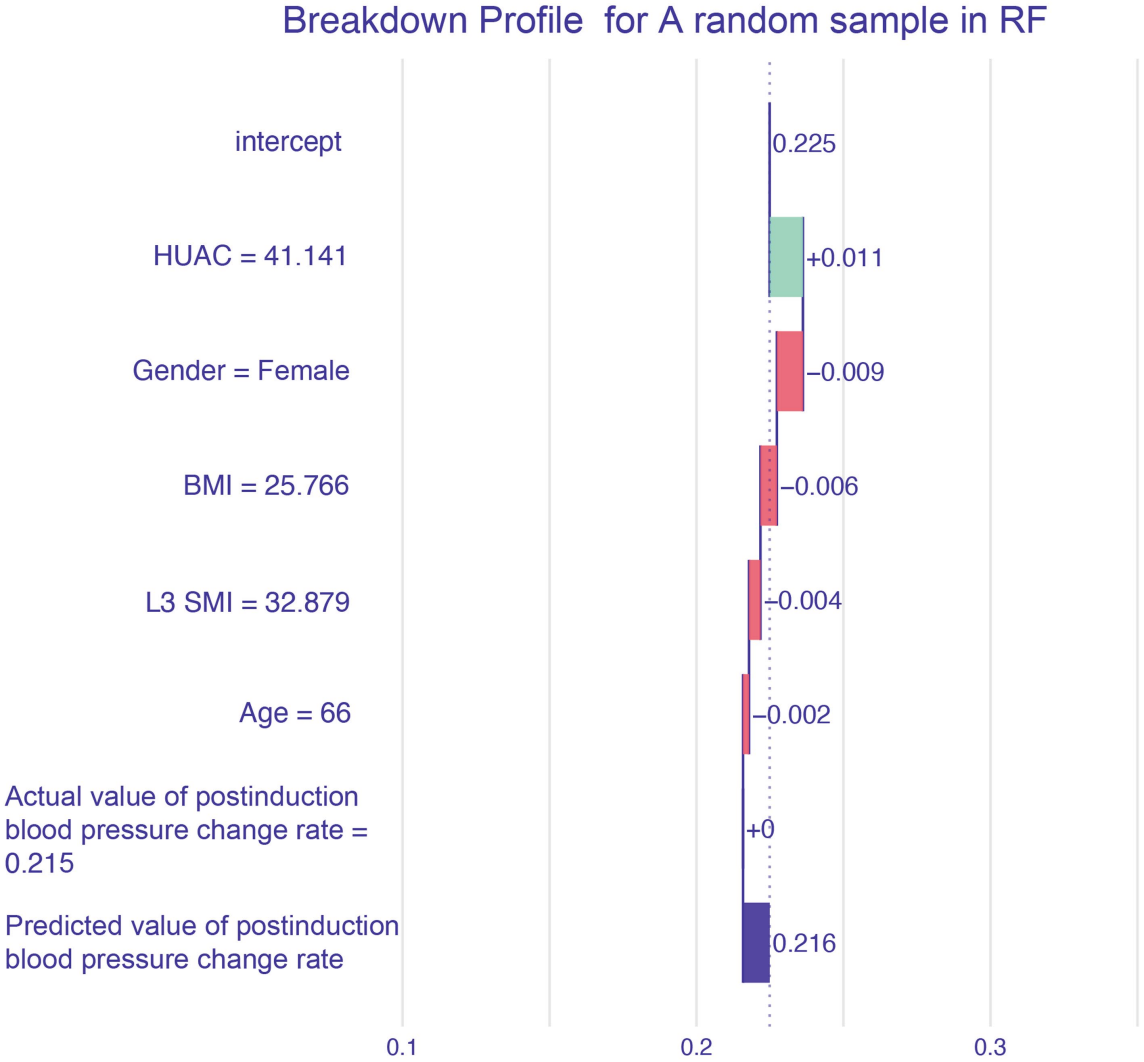
Breakdown profile.

The breakdown profile in Figure 6 shows the contribution of each variable to the prediction of a single sample. The model predicts that the value of a sample is 0.216 (the actual value of postinduction blood pressure change rate = 0.215), and the red and blue bars display the impact of each variable on the prediction. The predicted value was equal to the sum of the contributions of each feature.

## 4 Discussion

The effect of muscle mass in Chinese patients has not been thoroughly studied, particularly in patients with perioperative cancer. The complex patterns and relationships between malnutrition, cachexia, sarcopenia, and myosteatosis in cancer patients, among which sarcopenia and myosteatosis are highly prevalent, may lead to health-related adverse consequences, including PIH (20, 21). CT of the third section of the lumbar spine has been proven to be feasible and accurate for measuring body composition in patients with cancer. In particular, CT images of a specific lumbar vertebral landmark (L3) were significantly correlated with the overall muscles. However, due to the lack of unified cutoff values for sarcopenia and muscle steatosis in L3 SMI and HUAC, we used CT measurement data from L3 SMI and HUAC to predict postinduction blood pressure change rate (22). The sorting chart shows that HUAC and L3 SMI had the highest contributions to postinduction blood pressure change rate. HUAC and PIH were negatively correlated in the univariate partial dependence profile. When HUAC >35 or < 40, the postinduction blood pressure change rate curve decreases sharply and undergoes a qualitative change. The partial dependence plots of the L3 SMI and PIH showed curve fitting. When L3 SMI was <40 or > 55, the blood pressure after induction was lower than the baseline blood pressure. When L3 SMI >40 or < 55, the blood pressure after induction was equal to or higher than the baseline blood pressure. The mechanisms underlying muscle loss and degeneration and PIH may be chronic inflammation, imbalance in activity/reactivity of the sympathetic and parasympathetic divisions of the autonomic nervous system, and neurohumoral adaptations (23).

Previous studies have shown that patients with lower BMI have a higher incidence of PIH (24). However, from the partial dependence profile, BMI >18 or < 24 and BMI >24 or < 32 resulted in a slight decrease and increase in blood pressure, respectively, after induction. Moreover, when the patient’s BMI was >32, as the BMI increased, there was a significant decrease in blood pressure after induction. This may be related to the pharmacokinetics and TCI parameter settings of propofol. Previous research has shown that age is a risk factor for PIH. The mechanism of PIH may be related to aging, leading to structural changes in the arterial blood vessels, decreased cardiac contractility, insufficient relative circulating blood volume, and weakened hemodynamic regulation ability (25). From the partial dependence profile, age was positively correlated with the occurrence of postinduction blood pressure change rate; that is, the older the patient, the greater the likelihood of PIH occurrence. A systematic review described the relationship between sex and PIH, but the results were contradictory, with the male sex being a risk factor in one study and the female sex in another (26, 27). This study indicates that men are more likely to experience induced hypotension than women.

Hemodynamic instability frequently occurs during tumor resection. A growing body of evidence suggests that intraoperative hypotension is associated with adverse postoperative outcomes. Postoperative complications of hypotension include renal complications: postoperative acute kidney injury, cardiac complications (postoperative myocardial injury and infarction), gastrointestinal complications (ulcers and ischemic colitis), neurological complications (spinal cord or central nervous system ischemia and postoperative cognitive impairment), and mortality (28). PIH accounts for approximately one-third of all cases of perioperative hypotension. Compared with hypotension during surgery, PIH is mainly caused by the patient’s own state and anesthesia management, is not related to the surgical process, and can be prevented to a certain extent (29). However, relatively little research has been conducted on the risk factors for PIH. Based on the above reasons, it is particularly important to screen for postinduction blood pressure change rate risk factors and establish predictive models to provide scientific data for anesthesiologists to avoid a decrease in blood pressure after induction.

The selection of feature variables is an important part of modeling and is crucial in machine learning. This study used Boruta feature variable screening to achieve optimal and simplified subsets. The principle of the Boruta algorithm is to generate a “shadow attribute” for each variable and calculate the Z-score value for each variable using the RF model. When the Z-score was significantly higher than the highest shadow attribute value, the input variable was considered and retained as the dependent variable.

For machine learning, this study used the mlr3 ecosystem and its extension packages. The latest generation of R packages can be used for data preprocessing, pipelines, model fitting, selection, and visualization. After standardizing the data, repeated cross-validation and hyperparameter optimization were used to establish and select the optimal machine learning model in the training set, and external validation was conducted. A postinduction blood pressure change rate RF model with accurate predictions was obtained using multiple evaluation parameters of the regression model. When training the model, we selected multiple machine learning methods and used repetitive cross-validation and hyperparameter optimization to fit the data for each method. Repeated cross-validation is an extension of cross-validation that can achieve more stable and reliable model evaluation and reduce the impact of random factors on the model results. The generalizability of the model can be evaluated more fully by partitioning the training dataset multiple times. Second, the performance of the machine models is directly related to the hyperparameters. The better the hyperparameter adjustment, the better the resulting model. Finally, among the four candidate models, the optimal model with the lowest MSE was selected. During external validation, we used time-period validation and calculated multiple evaluation indicators of the regression model to verify the model’s robustness and generalizability (30).

The challenges of applying machine learning lie primarily in the lack of interpretability and repeatability of machine learning-generated results, which may limit their application. Interpretable machine learning can effectively open the “black box” of machine learning (31, 32). In this study, the degree of contribution of each feature variable was explained through an importance sorting chart, and the trend of the result variable changing with the feature variable was explained through a univariate partial dependency profile and visualization prediction of random individual samples through a breakdown profile. This solves the problem of lack of interpretability in predictive models, allowing clinical doctors to take timely intervention measures for high-risk PIH.

The study limitations may have affected the results. First, we included multiple clinical factors but did not include non-clinical factors, such as the patient’s psychological factors. Second, this study used time-series segmentation to achieve external validation. In the future, the model requires an independent dataset to test its extrapolation and generalizability.

In conclusion, this study used machine learning algorithms to predict the risk of PIH in 318 patients who underwent colorectal tumor resection under general anesthesia. We analyzed 3,498 data points, identified important feature variables, and established a postinduction blood pressure change rate model with acceptable predictive ability.

## Data availability statement

The raw data supporting the conclusions of this article will be made available by the authors, without undue reservation.

## Ethics statement

The studies involving humans were approved by the Ethics Committee of Shijitan Hospital. The studies were conducted in accordance with the local legislation and institutional requirements. The ethics committee/institutional review board waived the requirement of written informed consent for participation from the participants or the participants’ legal guardians/next of kin because this study is a single center retrospective study.

## Author contributions

WS: Formal analysis, Writing – original draft. DG: Writing – original draft, Conceptualization, Data curation. PL: Data curation, Methodology, Writing – review & editing. MS: Data curation, Methodology, Writing – review & editing. LL: Methodology, Formal analysis, Writing – review & editing. HM: Writing – review & editing. TL: Funding acquisition, Supervision, Writing – review & editing.
